# Effect of preparation design on fracture resistance of molars restored with occlusal veneers of different CAD-CAM materials: an in vitro study

**DOI:** 10.1186/s12903-024-04904-4

**Published:** 2024-10-01

**Authors:** Ahmed Ismail Taha, Mona Elshirbini Hafez

**Affiliations:** 1 Prosthodontic Department, Faculty of Dentistry, Kafr Al Sheikh University, Mubark Road, 33511 Kafr Abu Tabl, Kafrelsheikh Governorate, Kafr Al Sheikh, 6860404 Egypt; 2 Conservative Department, Faculty of Dentistry, Kafr Al Sheikh University, Kafr Al Sheikh, Egypt

**Keywords:** Occlusal veneers, Preparation design, Lithium disilicate, CAD-CAM, Fracture resistance

## Abstract

**Background:**

Occlusal veneer had been evaluated for mechanical properties using lithium disillicate. However, studies evaluating the mechanical properties of occlusal veneer with different preparation designs and ceramic materials are lacking. So, this in vitro study aimed to evaluate the fracture resistance of occlusal veneers with two designs fabricated from two different ceramic materials.

**Material and methods:**

Fourty mandibular third molars were distributed to 2 groups (*n* = 20) according to preparation design: group (O) anatomical occlusal reduction and group (OA) anatomical occlusal and 1 mm axial reduction. Each group was additionally subdivided into two subgroups (*n* = 10) according to ceramic materials; in subgroup X, lithium disilicate (e.max CAD, Ivoclar AG, Schaan, Liechtenstein) was used, and in subgroup S, zirconia-reinforced lithium silicate (ZLS) (Vita Suprinity, VitaZahnfabrik, Bad Säckingen, Germany) was used. All specimens were cemented with a light-cure resin cement (Choice 2, Bisco, Schaumburg, USA). 5000 thermocycles were applied to all specimens with both temperatures of 5 °C and 55 °C in two water baths; the dwell time was 30s at each bath, and the transfer time was 10s. Then all specimens were subjected to a fatigue simulation under dynamic loading of 200 N for 250,000 cycles. A universal testing machine (5500R/1123, Instron, Norwood, USA) was used to evaluate the fracture strength with a crosshead speed of 1 mm/min. All data were analyzed statistically by using a two-way ANOVA, and for some violations of assumptions, these results were compared with those obtained by the nonparametric test (Scheirer Ray Hare) (α = 0.05).

**Results:**

A statistically significantly higher fracture resistance in the ‘OA’ (3389 N) compared to the ‘O’ (2787 N) group regardless of the ceramic material (*P* < .001) and a statistically significantly higher fracture resistance in the ‘X’ (3295 N) compared to the ‘S’ (2881 N) regardless of the preparation design (*P* = .015).

**Conclusions:**

For occlusal veneers, all preparation designs and materials (such as Vita Suprinity and e.max CAD) had clinically acceptable fracture resistance values that were greater than the maximal biting forces. On the other hand, the e.max CAD with occlusal veneer, including axial reduction design, demonstrated the maximum fracture resistance value. Finally, no relationship between fracture strength and mode of failure was found.

## Clinical implications

Proper preparation design and the use of dental materials with high fracture resistance are key factors in extending the life of occlusal veneer restorations. The highest fracture resistance was demonstrated by lithium disilicate glass-ceramic occlusal veneers with axial preparation, which may increase the clinical longevity of teeth restored with occlusal veneers.

## Introduction

Nowadays, restorative dentistry is targeting preserving the tooth structure, and the inquiry for ceramic partial-veneer restorations is growing, thanks to the tooth structure preservation and esthetic outcome [1].

Loss of tooth structure can be caused by some other non-caries lesions, including erosion, abfraction, and fracture, which induce the destruction of hard tooth structure that is needed to be restored [2]. In addition, malformed teeth may also need restoration of tooth shape for biological, functional, esthetic, or social purposes [3].

Occlusal surface and functional cusps are predominantly affected by destruction [4], which can affect the vertical dimension of occlusion, esthetics, maxillo-mandibular relationship, and occlusal stabilization [5]. Survival of tooth and restoration are criticized for preserving remnant tooth structure [6].

The fracture resistance strength of the dental material has an essential role in raising the longevity of conservative restoration [1]. Moreover, with the development of new productive adhesive bonding techniques [6] and dental materials, there is a preference to select conservative treatment modalities over destructive treatments.

A less destructive and esthetic restorative substitute treatment such as partial coverage has been utilized presently, including inlays, onlays, and overlays [7]. Recently, occlusal veneers with no margin design have been used as a restoration for the function and anatomy of a flawed occlusal surface [8].

Many studies in the past few years have recommended the use of ceramic occlusal veneers as a standard treatment for worn teeth [9, 10]. Ceramic occlusal veneers fabricated by the CAD-CAM (computer-aided design-computer-aided manufacturing) system facilitate most of the technical difficulty of restoration construction [8]. Thanks to improvements in the CAD-CAM scanner and milling unit, better optical images and fine-detail restorations are produced [11].

Lithium disilicate is one of the most promising of these novel materials because of its good medium- and long-term survivability, strong mechanical strength, and great optical characteristics. This allows the use of the material as a monolithic restoration in posterior teeth as well as for the esthetic anterior sector, making it the gold standard material for posterior indirect restorations. Moreover, it showed reasonable biomechanical characteristics in posterior tooth restorations to withstand occlusal forces at minimum thickness values of 0.7 mm without affecting their strength [12, 13].

Several publications have reported on the remarkable fatigue resistance and fracture strength of ceramic overlays composed of monolithic lithium disilicate [10]. Three materials (composite resin, Lava Ultimate, and lithium disilicate CAD) were compared for fracture resistance in two thicknesses for ultrathin (0.3 mm) and thin (0.6 mm) occlusal veneers. For ultrathin occlusal veneers, lithium disilicate CAD showed unexpectedly better fracture strength when bonded to both enamel and enamel-dentin. Comparable results were observed for Lava Ultimate and composite resin [14]. According to Heck et al. [15], lithium disilicate CAD performed better than Empress CAD and Lava Ultimate in terms of longevity at veneer thicknesses of 0.3 to 0.5 mm.

The passion to combine the advantages of both zirconia (ZrO2) and lithium disilicate glass-ceramic zirconia-reinforced lithium silicate (ZLS) was introduced. Which is a suitable material for both anterior and posterior restorations thanks to its aesthetics and mechanical properties. The high content of glassy matrix gives the material esthetic properties, while zirconia in a proportion of 8–12% improves the mechanical properties [16]. Moreover, ZLS can be etched, which allows restorations to be cemented with adhesive systems that are different from zirconium oxide [17]. ZLS occlusal veneer was evaluated for fitting compared to hybrid ceramic; it showed larger marginal and internal discrepancies without significant difference [18].

When it comes to fatigue resistance, and fracture load, studies on the mechanical behaviour of occlusal veneers restoration, teeth have demonstrated that occlusal veneers composed of ZLS and lithium disilicate had greater fracture resistance than those composed of PMMA resin and ploymer -infiltrated ceramic [19, 20]. Using finite element analysis (FEA), the stress distribution of dental occlusal veneers was investigated. The materials that were investigated in this study were hybrid ceramic, lithium disilicate, ZLS, feldspathic, and high translucency zirconia. High translucency zirconia exhibited the highest stress concentration, as demonstrated by the data. Lithium disilicate, feldspathic, ZLS, and hybrid ceramics came next [21]. An ultrathin bonded posterior occlusal veneer under vertical loading was modelled in a recent study using FEA with a geometric sphere acting as an antagonist. The outcomes showed that ceramic materials and CAD-CAM composite resin can both be good materials [22]. A further investigation demonstrated that, in comparison to resin composite occlusal veneers, lithium disilicate had a larger concentration of tensile stress in the FEA data [23].

Simultaneously with the improvements in ceramics, there have been advances in bonding and luting agents, as well as techniques, that have allowed the long-lasting success of adhesive restorations to be equal to or greater than those of mechanical retention [24]. However, despite improvements in adhesion technology and ceramic materials, full-coverage crowns are still the most common restorative treatment option for posterior teeth [25].

The fracture resistance of occlusal veneers is considered one of the main elements that can affect the longevity of restoration [26]. In addition, the failure modes can be noticed. The mode of fracture allows a better understanding of the failure mechanism and the distribution of stresses and predicts the survival of different restorations under intraoral forces [27]. Fracture resistance may not only be influenced by the material used but also by the design of the preparation. Nevertheless, their effect on fracture resistance is controversial [28].

The present study was aimed at assessing the fracture resistance of molars restored with occlusal veneers using two different designs for preparation and fabricated from two different CAD-CAM ceramic materials. The null hypothesis implies that neither the preparation designs nor the materials used will affect the fracture strength of the occlusal veneer with a significant difference.

## Materials and methods

The present study was recognized by the ethical committee of the Faculty of Oral and Dental Medicine and Surgery, Kafrelsheikh University, Kafrelsheikh, Egypt (KFSIRB200–900). Providing power (80%), significance (5%), a confidence interval (95%), and the effect size (1.65), the resulting sample size was 9 for each group [1]. A total of 20 specimens were enclosed in each group. Forty mandibular third molars of relatively equal size were extracted for therapeutic objectives. All patients were informed and signed consent for using their extracted teeth.

The proportions of each tooth buccopalatally and mesiodistally were calibrated by a caliper. For the standardization objective, a 0.5 mm variation was believed satisfactory for each calibration. It has been reported that small differences in natural tooth dimensions do not cause any changes regarding fracture strength values in vitro [29].

The collected teeth were precisely investigated with 10 × magnification to verify that there were no caries, anatomical defects, or cracks. Calculus and soft tissue reminants were eleminated using a hand scaler and pumice prophylaxis. The specimens were stored in a 0.1% thymol solution at room temperature until the beginning of production. In order to replicate the periodontal ligament, first, the mesiodistal width at the mid-root region of each tooth was measured using a calliper. The teeth were dipped in melted wax, beginning two millimetres below the CEJ, to create a 0.2–0.3 mm-thick wax film surrounding the root. Using a calliper, the thickness of the root was measured again after immersion to verify the thickness of the wax coating (extra wax was cut off using a carver). The next step involved mounting each tooth to a level 2 mm below the cementoenamel junction using self-curing acrylic resin (Lucitone HIPA, Dentsply Sirona, Ballaigues, Switzerland). The teeth were taken out of the acrylic moulds, and the resin sockets and root surface were scraped clear of the wax. The melted wax space was filled with a regular-viscosity polyether impression (Elite P&P, Zhermack SpA, Polesine, Italy), and the roots were inserted back into their respective sockets right away. All the excess material was eliminated.

In accordance with occlusal veneer preparation design, selected teeth were randomly distributed into two groups (*n* = 20): group (O) occlusal reduction and group (OA) occlusal reduction with axial preparation ended with chamfer finish line. For the purpose of standardisation, a rubber base index for the crown was made before resuction to ensure the required thickness of preparation was reached. In addition, guiding grooves were used. The reduction of all teeth was performed by a skilled operator (A. T.) using a high-speed handpiece with a coolant system and equipped with a micromotor using coarse diamond (6856.FG.014, Komet, Rock Hill, United States) and under water irrigation according to the standard guidelines. The surfaces were refined with fine-grained diamonds (8856.FG.014, Komet, Rock Hill, United States).The burs were replaced with a new one after five times of usage. The design of the tooth preparation was as follows: Occlusal preparation of all cusps was performed anatomically to a depth of 1.5 mm for group (O). While, in group (OA), the tooth margins received an axial reduction of 1 mm in height that ended with a 1 mm chamfer finish line after occlusal preparation. Each group was additionally split into two subgroups (X) and (S) (*n* = 10), according to the CAD-CAM materials used. In subgroup (X), lithium disilicate (e.max CAD, Ivoclar AG, Schaan, Liechtenstein) was used, and in subgroup (S), ZLS (Vita Suprinity, VitaZahnfabrik, Bad Säckingen, Germany) was used.

**Fig. 1:**
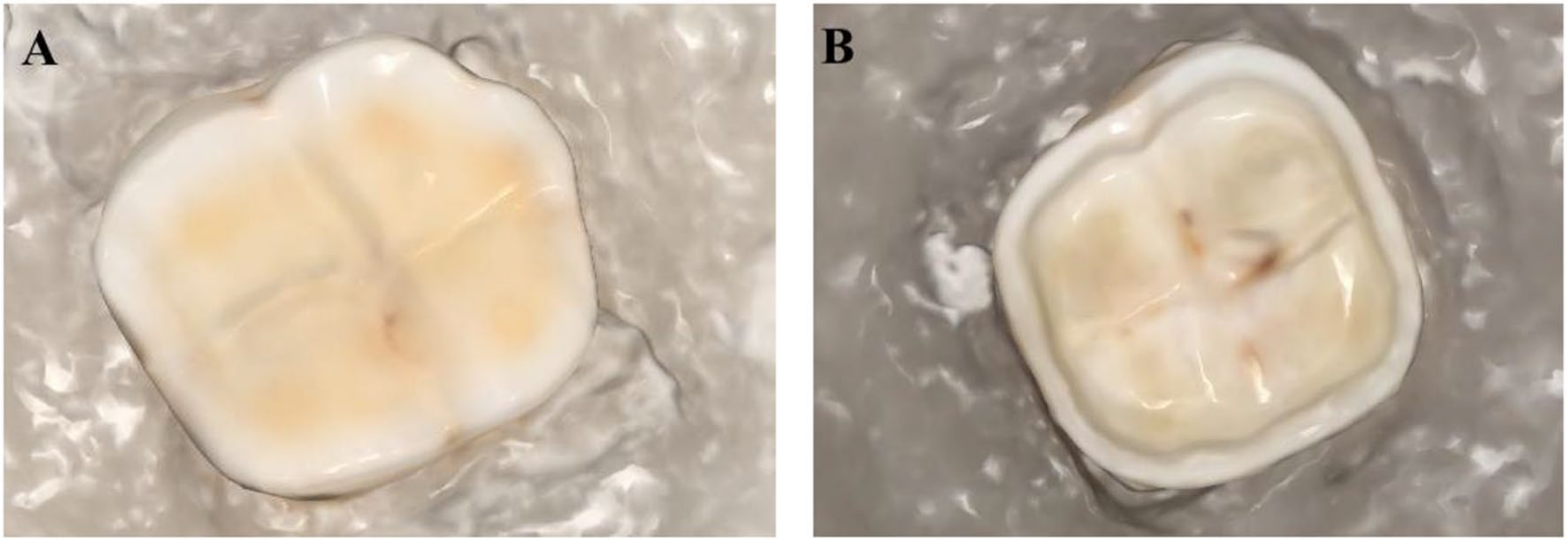
(**A**) scanned occlusal veneer preparation and (**B**) scanned occlusal veneer preparation with short axial reduction

After preparation of all molars, digital impressions were captured using an intraoral scanner (Medit i700, MEDIT Corp., Seoul, Republic of Korea), as shown in Fig. 1A–B. The exported standard tessellation language (STL) file was transferred to a software program (DentalCAD 3.0 Galway 2021, Exocad, Darmstadt, Germany) to design the occlusal veneer restoration according to the restoration design of each group. The restoration designs were sent to a 5-axis milling machine (Coritec 250i, imes-icore GmbH, Eiterfeld, Germany). For e.max CAD and vita Suprinity occlusal veneers, additional crystallisation was done using a furnace (Vita Vacumat 6000 M; VITA Zahnfabrik GmbH, Bad Säckingen, Germany) according to the manufacturer instructions.

Following milling, pressure sites on the prepared teeth were assessed for occlusal veneer restorations. These areas were detected using a water-soluble pressure-indicating paint (PIP; Keystone Industries, Singen, Germany). All pressure areas found were eliminated using a finishing green diamond tip (DCB, Schleifer, Komet Dental, Lemgo, Germany) until full seating was confirmed. Following try-in, the occlusal veneer restorations were glazed in accordance with the guidelines provided by the manufacturers for both materials.

The protocol of cementation was the same for both materials used according to the manufacturer’s instructions. The intaglio surfaces of the occlusal veneers were treated with hydrofluoric acid etch gel 4.5% (Porcelain etch, Ultradent Products, Cologne, Germany) for 20s, rinsed, and dried. Surfaces were coated with silane coupling agents (Silane, Ultradent Products, Cologne, Germany) for 1 min. For the tooth side, the prepared enamel tooth surfaces were treated with 37.5% phosphoric acid etch (Ultra-Etch, Ultradent Products, Cologne, Germany) for 15s, rinsed, and dried. The primer and bond system (FL-Bond II, Shofu Dental GmbH, Kyoto, Japan) was applied to the prepared tooth surface according to manufacturer instructions. The prime was applied to enamel and dentin and thinning within 20s, then the adhesive was applied to tooth structure and cured with light-cure for 15s.

**Fig. 2:**
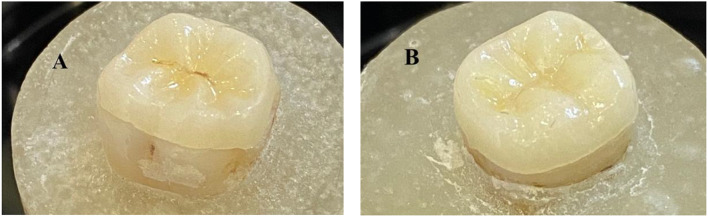
(**A**) Occlusal veneer after cementation for group (O) and (**B**) Occlusal veneer after cementation for group (OA)

Occlusal veneers were cemented with a light-cure resin luting cement (Choice 2, Bisco, Schaumburg, USA). To help with the restoration’s seating, steady finger pressure was used until any excess cement was released and removed. A specially made seating device was then used to apply a steady pressure of 0.5 kg. Each surface was subjected to a visible light cure (Bluephase G2, Ivoclar AG Schaan, Liechtenstein) for 20s following the removal of excess cement. After cementation of occlusal veneers as shown in Fig. 2A–B, all the specimens were reserved in distilled water at 37 °C for 1 week and then thermocycled for 5000 cycles with both temperatures of 5 °C and 55 °C in 2 water baths for 30s at each bath, and the shifting time was 10s. After thermocycling, a fatigue test was accomplished using a universal testing machine (5500R/1123, Instron, Norwood, USA) by exposing the restored teeth to a dynamic loading of 200 N with a metal rod 6 mm broad at 6 Hz of frequency.

**Fig. 3:**
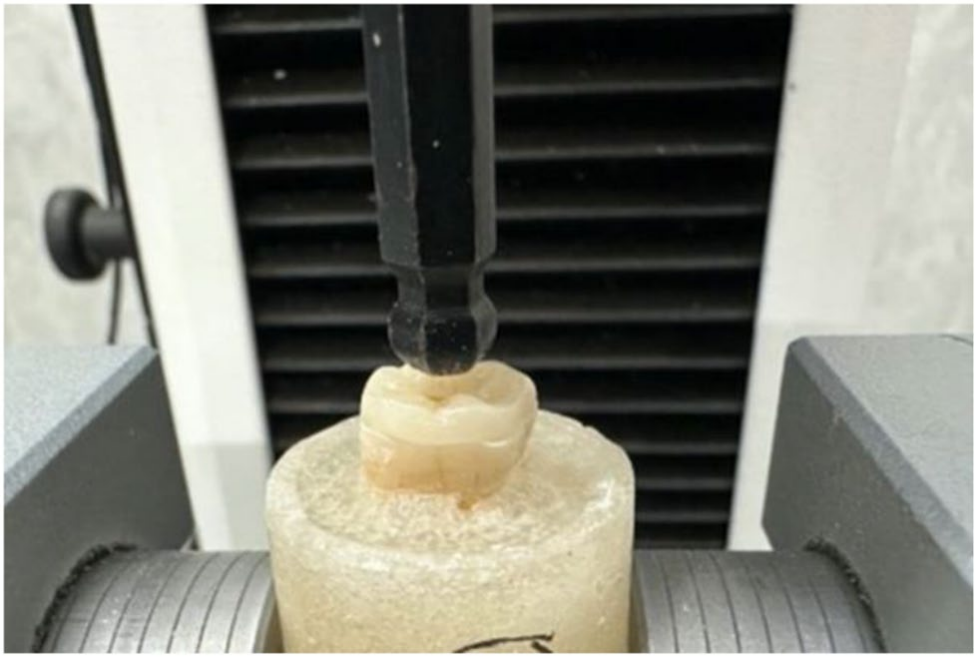
Fracture resistance test with universal testing machine

The specimens were submerged in distilled water at 37 °C for 250,000 cycles during this fatigue simulation. Immediately after the cyclic fatigue, the fracture resistance of the teeth was evaluated by a universal testing machine (5500R/1123, Instron, Norwood, USA) with a load cell of 5 KN. A round-end stainless steel rod with a 6 mm cross-section was used to apply load with a crosshead speed of 1 mm/min at the center of the line connecting the buccolingual cusps until fracture, as shown in Fig. 3.

### Fractographic analysis

All teeth were investigated under a stereo microscope (Nikon SMZ745T stereo microscope, Nikon Co., Tokyo, Japan) with 20× magnifications to evaluate failure mode, which was classified according to Burke’s classification into 4 categories [30]: (I) fracture within the restoration, (II) restoration fracture with a small part of tooth, (III) more than 50% of the tooth structure fractured without inclusion of root, and (IV) fracture across the restoration and tooth with inclusion of root. Additionally, fractured surfaces were examined with scanning electron microscope (SEM) (JSM- 6510 lv; JEOL, Tokyo, JAPAN) at magnification 250x to evaluate and illustrate the failure origin and characterize the fractographic features.

### Statistical analysis

Data was investigated using IBM-SPSS software (version 27, 2020). A 2-way ANOVA was administered to investigate the effects of preparation design and material on fracture resistance. To test for the assumptions of the two-way ANOVA, residual analysis was performed. Inspection of a boxplot was used to assess outliers; Shapiro-Wilk’s test was used to assess the normality for each cell of the design; and Levene’s test was used to assess the homogeneity of variances. For statistically nonsignificant interactions, the main effect of each factor was reported. For some violations of assumptions, these results were compared with those obtained by the nonparametric test (Scheirer Ray Hare). A Mann-Whitney U-test was used to compare fracture resistance between the two groups. The results were considered statistically significant if *P* ≤ .050 for any test used.

## Results

The descriptive statistics of fracture resistance (mean ± standard deviation) were demonstrated in Table 1.

**Table 1:** Descriptive statistics of fracture resistance (Newton)

Group	CAD-CAM material	Mean	±SD
‘O’	‘X’	2965.40	± 453.356
	‘S’	2608.70	± 578.896
‘OA’	‘X’	3625.80	± 553.512
	‘S’	3153.80	± 464.454

*Notes* SD = standard deviation

### Results of two-way ANOVA for fracture resistance (Newton)

There were outliers in all treatment combinations, particularly O/Z and OA/S. Residuals were normally distributed (*P* > .05) in all treatment combinations. There was homogeneity of variances (*P* = .961). The test was carried on, and the results were compared with those obtained by the nonparametric test.

There was no statistically significant interaction between group and CAD-CAM materials on fracture resistance, F(1, 36) = 0.125, *P* = .726, partial η^2^ = 0.003.

Therefore, an analysis of the main effects for both groups and CAD-CAM materials was performed.

Table 2 showed a statistically significantly higher fracture resistance in the ‘OA’ group than that of the ‘O’ group, regardless of the CAD-CAM material, and a statistically significantly higher fracture resistance in the ‘X’ than that of the ‘S’ CAD-CAM material, regardless of the group.

**Table 2:** Main effect of group and CAD-CAM materials on fracture resistance

Main effect	Mean	SE	F	Sig.	Partial η^2^
Group					
‘O’	2787.05	115.26	13.674	< 0.001	0.275
‘OA’	3389.80	115.26			
CAD-CAM material					
‘X’	3295.60	115.26	6.462	0.015	0.152
‘S’	2881.25	115.26			

*Notes* SE = standard error. Sig. = significance (p-value). Partial η^2^ is a measure of effect size

#### Results of scheirer ray hare data analysis tool

The assumptions are that the interaction groups must be equal-sized and contain at least five sample members. In our data, this assumption was met (the interaction groups are equal-sized, with 10 sample members per group). The test was done using the Real Statistics Data Analysis Tool (the Real Statistics Resource Pack in Excel). The row factor was the CAD-CAM material, while the column factor was the group. The null hypothesis for the Rows factor, namely that the two archwires are equally effective, is rejected (*P* = .045); the null hypothesis for the Columns factor, namely that the two groups are equally effective, is also rejected (*P* = .001); and the CAD-CAM material * group interaction was not statistically significant (*P* > .05).

Table 3 showed the results of the Scheirer Ray Hare Test for a two-way ANOVA on ranked data, which was run to assess the interaction and main effects of group and CAD-CAM material on fracture resistance. Like the results of a two-way ANOVA, there was no statistically significant interaction between group and CAD-CAM material on fracture resistance. The main effects of each factor were statistically significant.

**Table 3:** Scheirer ray hare test for two-way ANOVA on ranked data (fracture resistance)

*Test*	*SS*	*df*	*H*	*P-value*
Rows	547.6	1	4.006829	0.045
Columns	1392.4	1	10.18829	0.001
Interaction	1.6	1	0.011707	0.914
Within	3388.4	36		
Total	5330	39		

*Notes* df = degrees of freedom. SS = Sum of squares

**Fig. 4:**
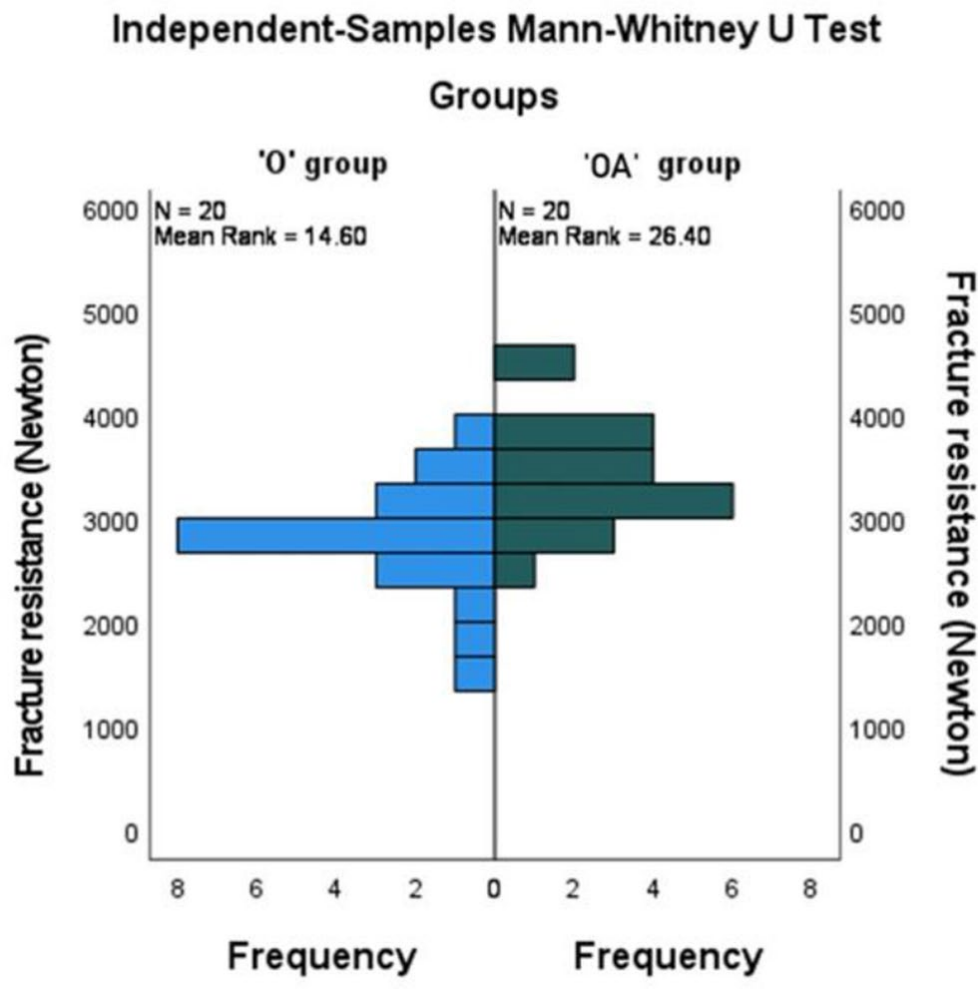
Mean ranks of fracture resistance in the two groups

Figure 4 showed a statistically significantly higher mean rank of fracture resistance in the ‘OA’ group compared to the ‘O’ group (*P* = .001). The median (Q1-Q3) was 2774 (2630.3–3105) in the ‘O’ group compared to 3367.5 (3021.5-3693.5) in the ‘OA’ group.

**Fig. 5:**
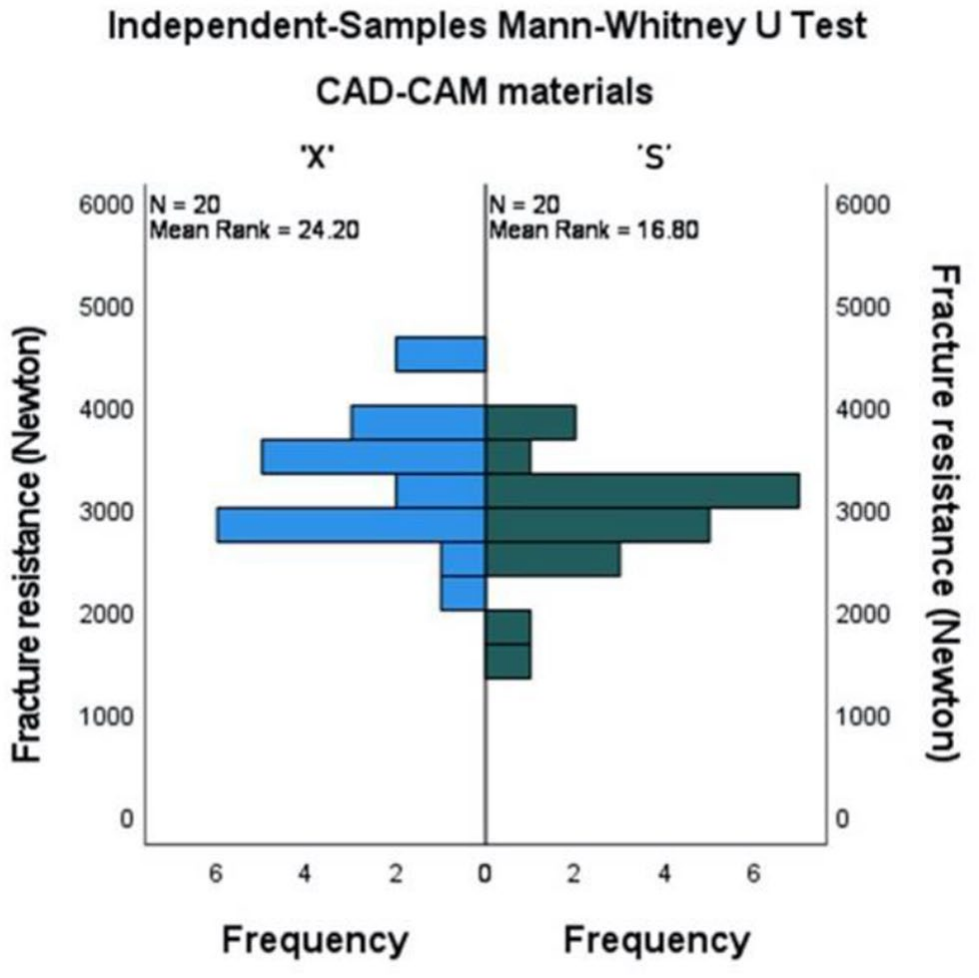
Mean ranks of fracture resistance in the two CAD-CAM materials

Figure 5 showed a statistically significantly higher mean rank of fracture resistance in ‘X’ material compared to ‘S’ material (*P* = .046). The median (Q1-Q3) was 3276.5 (2895-3668.5) in ‘X’ material compared to 2878.5 (2660.3-3244.8) for ‘S’ material.

### Failure mode analysis

Failure modes after the fracture resistance test for two groups were demonstrated in Table 4. The results showed that type III and type IV failure modes were the most common in both groups—about 80% of failure modes—and type II failure mode was the least common, while the specimens didn’t show any failure modes for type I.

**Table 4:** Mode of failures in different subgroups

groups	subgroups	Failure modes			
		I	II	III	IV
O	X	0	2	4	4
	S	0	2	4	4
OA	X	0	2	3	5
	S	0	1	4	5

**Fig. 6:**
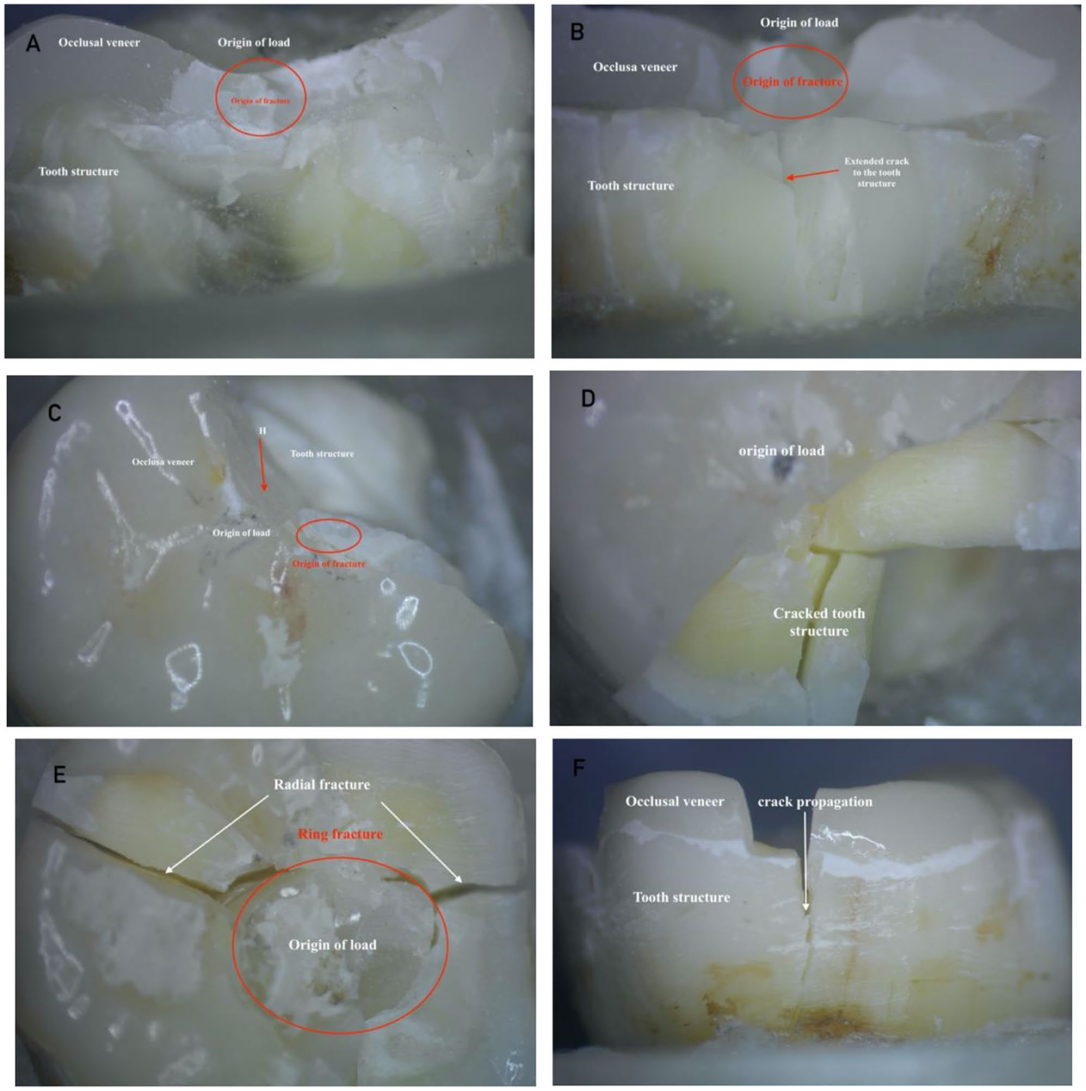
(**A**) stereomicroscope view of failure mode with x20 magnifications (**A** and **B**) failure mode type II, (**C** and **D**) failure mode type III and (**E** and **F**) failure mode type IV (showing origin of load, origin of fracture, and ring fracture)

**Fig. 7:**
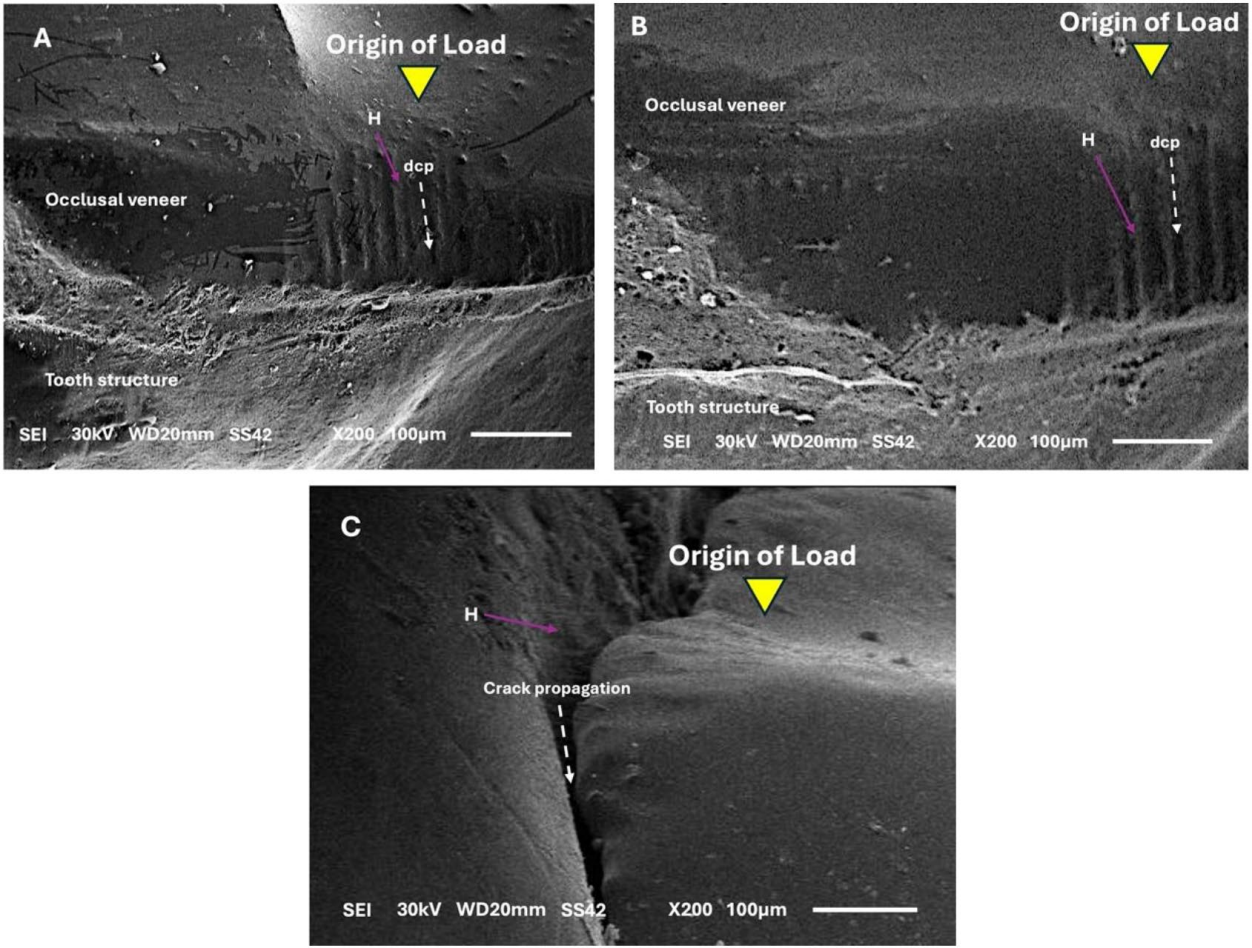
SEM view of fractured surface with magnification x200 showing (origin of load, H = hackle, and dcp = crack propagation direction) (**A**) failure mode type II, (**B**) failure mode type III and (**C**) failure mode type IV

The same failure patterns were revealed by the fractographic investigation for both restorative materials. Radial crack failures were seen in the lithium disilicate and ZLS occlusal veneers. Furthermore, a ring break was observed in the loading site’s central fossa in group (O). The fracture extended to the tooth structure, the crown’s edge, the groove, and the fossa. On the contact loading area of the occlusal surface, layered and imbricate fractures were observed as shown under stereomicroscope in Fig. 6A-F. According to SEM, these failures started at the loading site on the occlusal surface and moved towards the bonding surface. The direction of the crack’s propagation and its hackle lines were noticed as shown in Fig. 7A-C.

## Discussion

The results of current research stated that fracture resistance showed a significant difference between groups regardless of materials and significant differences in fracture resistance between materials regardless of preparation design. Thus, the null hypothesis was rejected.

At the present time, conservative restorative modalities have the primary interest of clinicians because they allow minimal tooth reduction with conservative preparation designs [31]. In turn, occlusal veneers emerged, and some in vitro studies reported that veneer restorations fabricated from high-strength ceramic materials produced predictable restorations for worn occlusal surfaces [20].

Unfortunately, there is little evidence about novel occlusal veneer restoration design compared with occlusal veneer, including axial preparation design. Accordingly, restorations with a minimal preparation design issue are still arguable, and it is essential to examine preparation designs midway between occlusal veneers and a full veneer crown.

Because the polyether impression behaves in a viscous and non-linear manner under external force, similar to the behaviour of the periodontal ligament, it was chosen for the current study. The elastic modulus of the Impregnum Soft Light Body was assessed in a different investigation, and it was discovered to be more similar to the human periodontal ligament [32].

The thickness of the occlusal veneer used in the present study was 1.5 mm, as it was stated in a previous study that thickness or more may be critical for posterior crowns milled from lithium disilicate. The same study presented that 1.5 mm occlusal thickness compared to 1.0 mm revealed a high significant difference in fracture resistance, which might be an important factor in the survival rate of ceramic crowns. But no significant difference was found between the restorations with thicknesses of 1.5 mm and 2.0 mm [33].

ZLS was used in the present research and compared to lithium disilicate, as it has been approved by several studies to have fracture resistance values higher than the physiological masticatory force ranges (230–698 N) [19]. Additionally, the zirconia fillers may act as an additional toughening mechanism [34]. Moreover, it is an etchable ceramic and exhibits high translucency [35].

In the current study, a light-cure resin luting cement (Choice 2, Bisco, Schaumburg, USA) with a primer system (FL-Bond II, Shofu Dental GmbH, Kyoto, Japan) was used for cementation. Recently, several forms of resin cement have been introduced, and the popularity of self-adhesive resin cement has increased because of its ease of application in one step. Furthermore, it has been demonstrated that its mechanical and adhesion features are much lower than those of conventional resin cements using three steps [36]. Accordingly, conventional resin cement was used to avoid much weaker values with self-adhesive resin cement.

The selected finish margin for the preparation was a chamfer, as it was well produced by the milling machine because of the round internal, and in addition, the burs can’t mill sharp angles. Different margin configurations for ceramic crowns were evaluated in a systematic review and found that the chamfer margin has better internal adaptation than that with a shoulder [37]. Additional research has evaluated the fracture resistance of CAD-CAM crowns with different finish line configurations and stated that crowns with a chamfer finish line afford resistance to fracture, which is higher than that for crowns with a shoulder finish line [38].

The current study simulated the temperature fluctuations of an oral cavity and six months of clinical care using thermocycling, which involved 5000 cycles at bath temperatures of 5 °C and 55 °C and dwell lengths of 20s [39]. In addition, the specimens were subjected to 250,000 cycles of fatigue simulation, which is equivalent to one clinical year of function [40].

The current study’s findings demonstrated that, regardless of the CAD-CAM material, occlusal veneers with axial preparation had a higher fracture resistance than those without (*P* < .001). This difference was statistically significant. The mean fracture resistance for both materials was found to be (2787 N) in group O and (3389 N) in group OA.The explanation is that the short axial walls with chamfer margin might have been resisting the shear stresses within the walls and better distributing the load across the margin, thus decreasing the load on the occlusal surface [41].

The results of previous research have been agreeable and have shown that preparation design has an effect on the fracture resistance of partial veneer restorations [42, 43]. But research studying the effect of preparation designs on an occlusal veneer is limited [1].

The result of the present study was not agreeable with Falahchai et al. [1], as they stated that most conservatively occlusal veneers prepared with an anatomical occlusal reduction resulted in higher fracture resistance than those prepartions that included a chamfer preparation. But both studies were in agreement that both preparation designs had satisfactory results for fracture resistance.

A recent study by Jurado et al. [44] reported that occlusal veneers with both preparation designs showed no significant difference in fracture strength. Another study by Jurado et al. [45]. stated that occlusal veneers with a margin had higher fracture resistance than those without a margin, and this result is consistent with the result of the present study.

The fracture resistance of ‘e.max CAD occlusal veneers’ (3295 N) showed a high statistically significant difference than the fracture resistance of ‘vita suprinity’ (2881 N) CAD-CAM material regardless of the group (*P* = .015). The inclusion of zirconia in the microstructure appears to increase material hardness, making it more susceptible to chipping during milling [46]. The adaptation of restoration can be compromised by chipping, which indirectly decreases fracture loads [47].

This result agreed with other research [48, 49] applied to different restorations, which stated that lithium disilicate occlusal veneers exhibit higher fracture resistance than ZLS. On the contrary, some other studies revealed the opposite results [50, 51].

Another study by El Ghoul et al. [52] examined the fracture resistance of endocrown, and the results revealed a higher fracture resistance for lithium sisilicate compared to ZLS, which is consistent with the result of the present study.

A fractured surface’s topography exhibits traditional crack patterns that are indicative of the material and related stress state. Understanding a portion, if not the all of the failure history, depends on the identification of these markers. The accurate replication of the cracked surface made it possible to identify classic features with high accuracy, which in turn revealed the direction in which the crack propagated and occasionally the fracture origin [53].

According to the results of this research, the restoration-cement-tooth complex showed evidence of a stronger bond than the applied force when crack formation without debonding form of failure occurred. Moreover, variations in preparation designs and materials used showed different variations of failure modes; nevertheless, there were no significant differences. It was found that in about 80% of the restoration failures noticed in this study for type III and IV, the tooth structure was fractured through the restorative material damage, which was agreeable with the previous studies [1, 8].

This might be as a result of the rigidity of both materials, their higher modulus of elasticity (100 GPa) than dentin, and the enormous stress concentrations they induce in crucial places, which could result in disastrous failures [54]. Failure mode investigation has been conducted to provide insight into the failure patterns and mechanical behaviour of ZLS restorations. It has been demonstrated that catastrophic and irreversible fracture patterns are the fundamental problem for silicate-based materials such as lithium disilicate and ZLS [55].

The limitations of this research are: firstly, the rearch was in vitro, which may vary from a clinical one; the intraoral scanning procedures would be more complicated because of saliva; and the accessibility of the scanner handling in the oral cavity. Secondly, the variation of the occlusal cusp angle of the crown and tooth substrate removal must be considered, as it results in different angulations of the prepared cusps that vary between the groups and affect the results. In addition, previous studies stated that the failure rate increased with a steep-angled cusp inclination compared to more flatter cusp angles [56]. Moreover, since extracted human molars were utilized, the age and size differences limit standardization of teeth [57]. Future research with a larger sample size and long-term in vivo studies is required to ensure the results of the present study.

## Conclusions

Under the limitations of this study.

- All materials used (lithium disilicate and ZLS) and preparation designs for occlusal veneer had clinically acceptable fracture resistance values that exceeded the maximum biting forces.
- Occlusal veneer with axial preparation had the highest fracture resistance values, no matter the materials used.
- Lithium disilicate occlusal veneers had the highest fracture resistance values, no matter the preparation design.
- There was no correlation discovered between fracture strength and manner of failure.

## Data Availability

The datasets generated and/or analysed during the current study are not publicly available due to [the research is not published yet] but are available from the corresponding author on reasonable reques.

## References

[1] Falahchai M, Babaee Hemmati Y, Neshandar Asli H, Rezaei E. Effect of tooth Preparation Design on Fracture Resistance of Zirconia-Reinforced Lithium Silicate Overlays. J Prosthodont. 2020;29:617–22. 32147878 10.1111/jopr.13160

[2] Nascimento MM, Gordan VV, Qvist V, Bader JD, Rindal DB, Williams OD, Gewartowski D, Fellows JL, Litaker MS, Gilbert GH, Dental Practice-Based Research Network Collaborative Group. Restoration of noncarious tooth defects by dentists in the Dental practice-based Research Network. J Am Dent Assoc. 2011;142:1368–75. 22130438 10.14219/jada.archive.2011.0138 PMC3229176

[3] Meade MJ, Dreyer CW. Tooth agenesis: an overview of diagnosis, aetiology and management. Jpn Dent Sci Rev. 2023;59:209–18. 37645267 10.1016/j.jdsr.2023.07.001 PMC10461125

[4] Demarco FF, Meireles SS, Sarmento HR, Dantas RV, Botero T, Tarquinio SB. Erosion and abrasion on dental structures undergoing at-home bleaching. Clin Cosmet Investig Dent. 2011;18:45–52. 10.2147/CCIDEN.S15943 PMC3652357 23674914

[5] Attin T, Filli T, Imfeld C, Schmidlin PR. Composite vertical bite reconstructions in eroded dentitions after 5·5 years: a case series. J Oral Rehabil. 2012;39:73–9. 21827523 10.1111/j.1365-2842.2011.02240.x

[6] Van Dijken JW, Hasselrot L. A prospective 15-year evaluation of extensive dentin–enamel‐bonded pressed ceramic coverages. Dent Mater. 2010;26:929–39. 20691334 10.1016/j.dental.2010.05.008

[7] Tsitrou E, Van Noort R. Minimal preparation designs for single posterior indirect prostheses with the use of the Cerec system. Int J Comput Dent. 2008;11:227–40. 19216314

[8] Johnson AC, Versluis A, Tantbirojn D, Ahuja S. Fracture strength of CAD/CAM composite and composite-ceramic occlusal veneers. J Prosthodont Res. 2014;58:107–14. 24636368 10.1016/j.jpor.2014.01.001

[9] Edelhoff D, Sorensen JA. Tooth structure removal associated with various preparation designs for posterior teeth. Int J Periodontics Restor Dent. 2002;22:241–9. 12186346

[10] Magne P, Schlichting LH, Maia HP, Baratieri LN. In vitro fatigue resistance of CAD/CAM composite resin and ceramic posterior occlusal veneers. J Prosthet Dent. 2010;104:149–57. 20813228 10.1016/S0022-3913(10)60111-4

[11] Schiffenhaus S. The non retentive ceramic overlay. A biomimetic alternative to the full coverage crown. Inside Dentistry. 2021;17:24–31.

[12] Luciano M, Francesca Z, Michela S, Tommaso M, Massimo A. Lithium disilicate posterior overlays: clinical and biomechanical features. Clin Oral Investig. 2020;2:841–8. 10.1007/s00784-019-02972-3 PMC13161036 31201516

[13] Yan J, Kaizer MR, Zhang Y. Load-bearing capacity of lithium disilicate and ultra-translucent zirconia. J Mech Behav Biomed Mater. 2018;88:170–5. 30173069 10.1016/j.jmbbm.2018.08.023 PMC6179910

[14] Valenzuela EBS, Andrade JP, da Cunha P, Bittencourt HR, Spohr AM. Fracture load of CAD/CAM ultrathin occlusal veneers luted to enamel or dentin. J Esthet Restor Dent. 2021;33:516–21. 32949221 10.1111/jerd.12658

[15] Heck K, Paterno H, Lederer A, Litzenburger F, Hickel R, Kunzelmann KH. Fatigue resistance of ultrathin CAD/CAM ceramic and nanoceramic composite occlusal veneers. Dent Mater. 2019;35:1370–7. 31351578 10.1016/j.dental.2019.07.006

[16] Bajraktarova-Valjakova E, Korunoska-Stevkovska V, Kapusevska B, Gigovski N, Bajraktarova-Misevska C, Grozdanov A. Contemporary Dental Ceramic materials, a review: Chemical Composition, Physical and Mechanical Properties, indications for Use. Open Access Maced J Med Sci. 2018;24:1742–55. 10.3889/oamjms.2018.378 PMC6182519 30338002

[17] Komar D, Bago I, Negovetić Vranić D, Kranjčić J, Brkić B, Carek A. Influence of different surface pretreatments of Zirconium Dioxide Reinforced Lithium Disilicate ceramics on the Shear Bond Strength of Self-Adhesive Resin Cement. Acta Stomatol Croat. 2021;55:264–79. 34658373 10.15644/asc55/3/4 PMC8514231

[18] Elbadawy AA, Omar EA, AbdElaziz MH. MicroCT evaluation for CAD/CAM occlusal veneer fit using two materials and three cement space settings. Braz Dent J. 2022;33:71–8. 10.1590/0103-6440202204764 PMC9645188 36043571

[19] Al-Akhali M, Chaar MS, Elsayed A, Samran A, Kern M. Fracture resistance of ceramic and polymer-based occlusal veneer restorations. J Mech Behav Biomed Mater. 2017;74:245–50. 28633093 10.1016/j.jmbbm.2017.06.013

[20] Al-Akhali M, Kern M, Elsayed A, Samran A, Chaar MS. Influence of thermomechanical fatigue on the fracture strength of CAD-CAM-fabricated occlusal veneers. J Prosthet Dent. 2019;121:644–50. 30711291 10.1016/j.prosdent.2018.07.019

[21] Tribst JPM, Dal Piva AMO, Penteado MM, Borges ALS, Bottino MA. Influence of ceramic material, thickness of restoration and cement layer on stress distribution of occlusal veneers. Braz Oral Res. 2018;32:e118. 30517427 10.1590/1807-3107bor-2018.vol32.0118

[22] Magne P, Stanley K, Schlichting L. Modeling of ultrathin occlusal veneers. Dent Mater. 2012;28:777–82. 22575740 10.1016/j.dental.2012.04.002

[23] Velho HC, Dapieve KS, Grassi EDA, Borges ALS, de Melo Marinho RM, Pereira GKR, Venturini AB, Valandro LF. Fatigue behavior, failure mode, and stress distribution of occlusal veneers: influence of the prosthetic preparation cusp inclinations and the type of restorative material. Clin Oral Investig 2023;27:5539-48. 10.1007/s00784-023-05173-1 37490118

[24] Perdigão J, Araujo E, Ramos RQ, Gomes G, Pizzolotto L. Adhesive dentistry: current concepts and clinical considerations. J Esthet Restor Dent. 2021;33:51–68. 33264490 10.1111/jerd.12692

[25] Ferraris F, Sammarco E, Romano G, Cincera S, Marchesi G. Comparison of posterior indirect adhesive restorations (PIAR) with different preparation designs according to the adhesthetics classification. Part 1: effects on the fracture resistance. Int J Esthet Dent. 2021;10:144–67. 33969972

[26] Kelly J, Giordano R, Pober R, Cima M. Fracture surface analysis of dental ceramics: clinically failed restorations. J Prosthet Dent. 1990;3:430–40. 2088380

[27] Preis V, Behr M, Hahnel S, Rosentritt M. Influence of cementation on in vitro performance, marginal adaptation and fracture resistance of CAD/CAM-fabricated ZLS molar crowns. Dent Mater. 2015;31:1363–9. 26345998 10.1016/j.dental.2015.08.154

[28] Elsaka SE, Elnaghy AM. Mechanical properties of zirconia reinforced lithium silicate glass-ceramic. Dent Mater. 2016;32:908–14. 27087687 10.1016/j.dental.2016.03.013

[29] Rosentritt M, Plein T, Kolbeck C, Behr M, Handel G. In vitro fracture force and marginal adaptation of ceramic crowns fixed on natural and artificial teeth. Int J Prosthodont. 2000;13:387–91. 11203659

[30] Burke F. The effect of variations in bonding procedure on fracture resistance of dentin-bonded all-ceramic crowns. Quintessence Int. 1995;26:293–300. 7568750

[31] Chen YW, Raigrodski AJ. A conservative approach for treating young adult patients with porcelain laminate veneers. J Esthet Restor Dent. 2008;20:223–36. 18767994 10.1111/j.1708-8240.2008.00184.x

[32] Rathi A, Chowdhry P, Kaushik M, Reddy P, Roshni, Mehra N. Effect of different periodontal ligament simulating materials on the incidence of dentinal cracks during root canal preparation. J Dent Res Dent Clin Dent Prospects. 2018;12:196–200. 30443305 10.15171/joddd.2018.030 PMC6231149

[33] Dhima M, Carr AB, Salinas TJ, Lohse C, Berglund L, Nan KA. Evaluation of fracture resistance in aqueous environment under dynamic loading of lithium disilicate restorative systems for posterior applications. Part 2. J Prosthodont. 2014;23:353–7. 24417233 10.1111/jopr.12124

[34] Chen XP, Xiang ZX, Song XF, Yin L, Machinability. Zirconia-reinforced lithium silicate glass ceramic versus lithium disilicate glass ceramic. J Mech Behav Biomed Mater. 2020;101:103435. 31586883 10.1016/j.jmbbm.2019.103435

[35] Sen N, Us YO. Mechanical and optical properties of monolithic CAD-CAM restorative materials. J Prosthet Dent. 2018;119:593–9. 28781072 10.1016/j.prosdent.2017.06.012

[36] Maravić T, Mazzitelli C, Mancuso E, Del Bianco F, Josić U, Cadenaro M, Breschi L, Mazzoni A. Resin composite cements: current status and a novel classification proposal. J Esthet Restor Dent. 2023;35:1085–97. 36924395 10.1111/jerd.13036

[37] Yu H, Chen YH, Cheng H, Sawase T. Finish-line designs for ceramic crowns: a systematic review and meta-analysis. J Prosthet Dent. 2019;122:22–30. 30782459 10.1016/j.prosdent.2018.10.002

[38] Alzahrani AM, Beyari AM, Emam ZN. The influence of the cervical finish line designs on the fracture resistance of CAD/CAM monolithic zirconia crowns, an in vitro study. Int J Health Sci Res. 2018;8:101–10.

[39] Eliasson ST, Dahl JE. Effect of thermal cycling on temperature changes and bond strength in different test specimens. Biomater Investig Dent. 2020;7:16–24. 10.1080/26415275.2019.1709470 PMC7033714 32128509

[40] Seydler B, Rues S, Müller D, Schmitter M. In vitro fracture load of monolithic lithium disilicate ceramic molar crowns withdifferent wall thicknesses. Clin Oral Investig. 2014;18:1165–71. 10.1007/s00784-013-1062-8 23904173

[41] Fages M, Bennasar B. The endocrown: a different type of all-ceramic reconstruction for molars. J Can Dent Assoc. 2013;79:d140. 24309044

[42] Oyar P, Durkan R. Effect of cavity design on the fracture resistance of zirconia onlay ceramics. Niger J Clin Pract. 2018;21:687–91. 29888712 10.4103/njcp.njcp_424_17

[43] Harsha MS, Praffulla M, Babu MR, Leneena G, Krishna TS, Divya G. The Effect of Cavity Design on Fracture Resistance and failure pattern in monolithic Zirconia partial Coverage restorations - an in vitro study. J Clin Diagn Res. 2017;11:ZC45–8. 10.7860/JCDR/2017/25305.9856 PMC5483808 28658906

[44] Jurado CA, Lee D, Ramirez P, Cortes-Treviño DA, Tsujimoto A. Fracture resistance of Chairside CAD/CAM Lithium Disilicate-reinforced ceramic Occlusal Veneers with and without margin and full Coverage crowns. Oper Dent. 2024;49:84–90. 38058016 10.2341/23-043-L

[45] Jurado CA, Tsujimoto A, Molisani J, Fu CC, Sadid-Zadeh R. Fracture resistance of chairside CAD-CAM lithium disilicate occlusal veneer with various designs after mechanical aging. J Prosthodont. 2024;6:1–6. 10.1111/jopr.13852 38706398

[46] Ramos Nde C, Campos TM, Paz IS, Machado JP, Bottino MA, Cesar PF, Melo RM. Microstructure characterization and SCG of newly engineered dental ceramics. Dent Mater. 2016;32:870–8. 27094589 10.1016/j.dental.2016.03.018

[47] Rojpaibool T, Leevailoj C. Fracture resistance of Lithium Disilicate ceramics Bonded to Enamel or dentin using different Resin Cement types and Film thicknesses. J Prosthodont. 2017;26:141–9. 26505488 10.1111/jopr.12372

[48] Kashkari A, Yilmaz B, Brantley WA, Schricker SR, Johnston WM. Fracture analysis of monolithic CAD-CAM crowns. J Esthet Restor Dent. 2019;31:346–52. 30821101 10.1111/jerd.12462

[49] Nishioka G, Prochnow C, Firmino A, Amaral M, Bottino MA, Valandro LF. Renata Marques De M. Fatigue strength of several dental ceramics indicated for CAD-CAM monolithic restorations. Braz Oral Res. 2018;11:e53. 10.1590/1807-3107bor-2018.vol32.0053 29898029

[50] Hamza TA, Sherif RM. Fracture resistance of monolithic Glass-ceramics Versus Bilayered Zirconia-based restorations. J Prosthodont. 2019;28:e259–64. 29044828 10.1111/jopr.12684

[51] Schwindling FS, Rues S, Schmitter M. Fracture resistance of glazed, full-contour ZLS incisor crowns. J Prosthodont Res. 2017;61:344–9. 28111135 10.1016/j.jpor.2016.12.008

[52] El Ghoul W, Özcan M, Silwadi M, Salameh Z. Fracture resistance and failure modes of endocrowns manufactured with different CAD/CAM materials under axial and lateral loading. J Esthet Restor Dent. 2019;31:378–87. 31067007 10.1111/jerd.12486

[53] Scherrer SS, Quinn JB, Quinn GD, Kelly JR. Failure analysis of ceramic clinical cases using qualitative fractography. Int J Prosthodont. 2006;19:185–92. 16602369

[54] Zhu J, Rong Q, Wang X, et al. Influence of remaining tooth structure and restorative material type on stress distribution in endodontically treated maxillary premolars: a finite element analysis. J Prosthet Dent. 2017;117:646–55. 27881319 10.1016/j.prosdent.2016.08.023

[55] Furtado de Mendonca A, Shahmoradi M, Gouvˆea CVD, De Souza GM, Ellakwa A. Microstructural and mechanical characterization of CAD/CAM materials for monolithic dental restorations. J Prosthodont. 2019;28:e587–94. 30121945 10.1111/jopr.12964

[56] Shahmoradi M, Wan B, Zhang Z, Wilson T, Swain M, Li Q. Monolithic crowns fracture analysis: the effect of material properties, cusp angle and crown thickness. Dent Mater. 2020;36:1038–51. 32534794 10.1016/j.dental.2020.04.022

[57] Fernández-Estevan L, Millan-Martínez D, Fons-Font A, Agustín-Panadero R, Román-Rodríguez JL. Methodology in specimen fabrication for in vitro dental studies: standardization of extracted tooth preparation. J Clin Exp Dent. 2017;9:e897–900. 28828157 10.4317/jced.54020 PMC5549588

